# Supplementary material for: Effectiveness of four antiretroviral regimens for treating people living with HIV
Source: PLoS One. 2020 Sep 28;15(9):e0239527. doi: 10.1371/journal.pone.0239527 (PMC7521729; doi:10.1371/journal.pone.0239527)
Supplement: Attachment — Submitted filename: Comments.docx [file pone.0239527.s001.docx]

This paper has very useful and important information. Authors explored multiple outcomes from observation cohort study among people living with HIV. Although it has useful information, it needs some improvements.

*General comment:*

*The paper will benefit language revision specifically in the way results were presented.*

*Check for consistencies of decimals in the tables*

*Avoid some medical terminologies where possible*

*Check for type errors*

*Specifics comments*

*Analysis section*

*Authors stated that “Individuals who changed regimens before completing 28 days of treatment were excluded” What was the criteria for the 28 days?*

Results:

**Virological response**

“When comparing participants who changed regimens during follow-up with

those who remained on the same regimen, we observed different survival curves (Fig 3,

3.a and 3.b). There was no difference in the virologic response between the groups.

There was a difference when we compared the participants who changed the regimen

during follow-up period with those who did not (p <0.001) (Fig 3.c).”

*This paragraph makes it difficult to follow: The way it is written it is not exactly what these graphs were demonstrating.*

*3a: compared differences of regimens among those who did not change treatment*

*3b. compared differences of regimens among those who changed treatment*

*3c. Compared survival between those who changed and those who did not change treatment. This section needs to be re-written*

Tables:

*(IC 95%), can this be written as 95% CI, this is standard*

*Check for typo error (table 1 Cliical situation*

*Check for the consistency of decimal places table 3.*

*3.4 (1.02-11.13) to 3.40, since you are doing 2 decimals through out.*

For this paragrapgh “After the multivariate analysis, the characteristics associated with a virologic

response were: being female; living with a family member/partner; being asymptomatic

when initiating ART; not report of adherence failure and initiating ART with a CD4

mm3 count of between 200-350 cells/”

*Would it be better to express those factors that were associated with increased or decreased likeli-hood of viral response separately?*

**Immunologic response**

Authors described cohort for each outcome been assessed For example

“To analyze the immunologic response, the sample consisted of 535 participants,

out of a total of 555 observations. Of the total participants, 82.5%

presented mm3 immunologic response (an increase of at least 50 cells/

of CD4 T lymphocytes) within up to 15 months of treatment. Most were male (62.8%), with a mean age of 39.2years (± 10.6), who had attended school for more than 9 years (65.7%), and 23.4%presented with comorbidities and other sexually transmitted infections. More than half

of the participants (52.9%) presented some symptoms suggestive of AIDS on initiating

ART. The median CD4 count before initiating ART was 281.5 (104; 420.5) with an

average of 305.2 cells. Most individuals (57.6%) initiated ART with an initial CD4 cell

count below 350, 18.3% were between 350 and 500 and 20.7% with a CD4> 500. At the

end of follow-up, 227 participants (42.4%) presented CD4 counts >500. Treatment

adherence failure was recorded in 17.8% of the participants”

*If the table for these characteristics is available, there is no need to describe a cohort for each outcome measure*

*Legend for table 3, authors reported gross odd ratio, it would be better to just state an adjusted instead of gross*

*Below information is also found in the results section. This information should be in the analysis section*

“For the multivariate analysis, the following variables were included in the

model: age group, adherence failure and initial CD4 cell count, with p <0.10 in the

multivariate analysis to verify association with immune response”

**Clinical failure.**

Incidence proportion should not be incidence rate

Below information can be better presented

“Amongst the participants who initiated ART with a CD4 count <200, 29.9% were

hospitalized during follow-up, whereas in those with higher counts, the proportion was

18.4%, with a significant difference for 1 year”

*This can be written as if one says “the incidence of hospitalization among those with CD4 < 200 c/ml was significantly higher compared to those with Cd4 count >= 200. (p< 0.001)”. something like that*

(13, 9%) should be 13.9%

**Discussion**

Terminologies such as “myelotoxicity” *Would avoid medical terminology and make it easier bone marrow suppression*

Figures:

Needs to be improved. Groups compared are hardly visualized, poor quality. Can customize analytical codes and produce high quality figures
