# Supplementary material for: Effectiveness of four antiretroviral regimens for treating people living with HIV
Source: PLoS One. 2020 Sep 28;15(9):e0239527. doi: 10.1371/journal.pone.0239527 (PMC7521729; doi:10.1371/journal.pone.0239527)
Supplement: Attachment — Submitted filename: Response to Reviewers.docx [file pone.0239527.s002.docx]

**Response to Reviewers**

Reviewer #1: This is a very comprehensive and interesting manuscript.

**Why the authors decided to sample from the retrospective cohort, and not use all the available data?**

The retrospective component is justified by the possibility of a longer follow-up of patients, allowing the study of outcomes that occur less early.

**It is not clear if the women who changed ART in the first year due to pregnancy are accounted in the "losses". "Women were excluded who had modified", revise language.**

Revised

**It is not clear what the 898 observations are as opposed to the number of patients (840).**

Second paragraph of Method- data analysis:

“ Follow-up was discontinued for those who changed to ART regimens other than those in the study groups, while those who changed to one of the studied regimens were considered as new exposure and the outcomes were independently observed.”

**Figure 3 a and b: give more details in the text about the difference.**

When comparing regimens in the group of participants who remained on the same regimen during the follow-up, there was no differences between the survival curves for virological response by regimens (Fig 3.a). The same we did found for those group who changed their regimen during the follow-up. (Fig 3.b). When comparing the survival curves of the virological response between these two groups of participants, we observed differences in their survival curves (p<0.001) (Fig 3.c).

Reviewer #2: This paper has very useful and important information. Authors explored multiple outcomes from observation cohort study among people living with HIV. Each section of the manuscript has been explored rigorously. The only concern is the English. The manuscript needs language revision.

Revised

The paper will benefit language revision specifically in the way results were presented.

Revised

Check for consistencies of decimals in the tables

Revised

Avoid some medical terminologies where possible

Revised

Check for type errors

Revised

**Authors stated that “Individuals who changed regimens before completing 28 days of treatment were excluded” What was the criteria for the 28 days?**

We considered this time criteria, because it was the habitual period they returned to the health service to receive their medication.

**Virological response**

**“When comparing participants who changed regimens during follow-up with**

**those who remained on the same regimen, we observed different survival curves (Fig 3,**

**3.a and 3.b). There was no difference in the virologic response between the groups.**

**There was a difference when we compared the participants who changed the regimen**

**during follow-up period with those who did not (p <0.001) (Fig 3.c).”**

**This paragraph makes it difficult to follow: The way it is written it is not exactly what these graphs were demonstrating.**

**3a: compared differences of regimens among those who did not change treatment**

**3b. compared differences of regimens among those who changed treatment**

**3c. Compared survival between those who changed and those who did not change treatment. This section needs to be re-written**

Revised

**Tables:**

**(IC 95%), can this be written as 95% CI, this is standard**

**Revised**

**Check for typo error (table 1 Cliical situation**

Revised

**Check for the consistency of decimal places table 3.**

**3.4 (1.02-11.13) to 3.40, since you are doing 2 decimals through out.**

Revised

**For this paragrapgh “After the multivariate analysis, the characteristics associated with a virologic response were: being female; living with a family member/partner; being asymptomatic when initiating ART; not report of adherence failure and initiating ART with a CD4 mm3 count of between 200-350 cells/”**

Revised

**Would it be better to express those factors that were associated with increased or decreased likeli-hood of viral response separately?**

We chose to present the multivariate analysis separately to facilitate the exposure of the data in more detail.

**Legend for table 3, authors reported gross odd ratio, it would be better to just state an adjusted instead of gross**

Revised

**Below information is also found in the results section. This information should be in the analysis section**

**“For the multivariate analysis, the following variables were included in the**

**model: age group, adherence failure and initial CD4 cell count, with p <0.10 in the**

**multivariate analysis to verify association with immune response”**

Revised

**Clinical failure.**

**Incidence proportion should not be incidence rate**

Revised

**Below information can be better presented**

**“Amongst the participants who initiated ART with a CD4 count <200, 29.9% were**

**hospitalized during follow-up, whereas in those with higher counts, the proportion was 18.4%, with a significant difference for 1 year”**

This can be written as if one says “the incidence of hospitalization among those with CD4 < 200 c/ml was significantly higher compared to those with Cd4 count >= 200. (p< 0.001)”. something like that (13, 9%) should be 13.9%

Revised

**Discussion**

Terminologies such as “myelotoxicity” Would avoid medical terminology and make it easier bone marrow suppression

Revised

**Figures:**

**Needs to be improved. Groups compared are hardly visualized, poor quality. Can customize analytical codes and produce high quality figures**

Revised
